# Intraoperative radiotherapy during awake craniotomies: preliminary results of a single-center case series

**DOI:** 10.1007/s10143-022-01838-9

**Published:** 2022-07-26

**Authors:** K. Steininger, K. H. Kahl, I. Konietzko, C. Wolfert, S. Motov, P. E. Krauß, T. Bröcheler, M. Hadrawa, B. Sommer, G. Stüben, E. Shiban

**Affiliations:** 1 Department of Neurosurgery, University Hospital Augsburg, Stenglinstraße 2, 86156 Augsburg, Germany; 2 Department of Radiation Therapy, University Hospital Augsburg, Augsburg, Germany; 3 Department of Anesthesia, University Hospital Augsburg, Augsburg, Germany

**Keywords:** Eloquently located brain tumor, Awake craniotomy, Intraoperative radiotherapy, Preliminary results

## Abstract

Awake craniotomies are performed to avoid postoperative neurological deficits when resecting lesions in the eloquent cortex, especially the speech area. Intraoperative radiotherapy (IORT) has recently focused on optimizing the oncological treatment of primary malignant brain tumors and metastases. Herein, for the first time, we present preliminary results of IORT in the setting of awake craniotomies. From 2021 to 2022, all patients undergoing awake craniotomies for tumor resection combined with IORT were analyzed retrospectively. Demographical and clinical data, operative procedure, and treatment-related complications were evaluated. Five patients were identified (age (mean ± standard deviation (SD): 65 ± 13.5 years (y)). A solid left frontal metastasis was detected in the first patient (female, 49 y). The second patient (male, 72 y) presented with a solid metastasis on the left parietal lobe. The third patient (male, 52 y) was diagnosed with a left temporoparietal metastasis. Patient four (male, 74 y) was diagnosed with a high-grade glioma on the left frontal lobe. A metastasis on the left temporooccipital lobe was detected in the fifth patient (male, 78 y). After awake craniotomy and macroscopic complete tumor resection, intraoperative tumor bed irradiation was carried out with 50 kV x-rays and a total of 20 Gy for 16.7 ± 2.5 min. During a mean follow-up of 6.3 ± 2.6 months, none of the patients developed any surgery- or IORT-related complications or disabling permanent neurological deficits. Intraoperative radiotherapy in combination with awake craniotomy seems to be feasible and safe.

## Introduction

The extent of primary resection of supratentorial glioma or metastasis correlates positively with progression-free and overall survival [2]. However, complete resection of tumor mass in the eloquent cortex, especially in the dominant hemisphere, represents a significant challenge. In these cases, awake craniotomies are performed to avoid postoperative neurological deficits mainly when language areas are at risk [16]. The initiation of the Stupp protocol in 2005 validated that the combination of high-grade glioma gross total resection and adjuvant radiotherapy positively increases overall and progression-free survival [8, 15]. In symptomatic single brain metastases, treatment options include surgical resection and/or adjuvant radiotherapy with no level 1 evidence regarding the superiority of one modality [6].

Recently, IORT, defined as the delivery of a precise dose of radiation to the tumor bed during surgery, has come into focus to optimize the oncological treatment of primary malignant brain tumors and metastases. By this method, a higher dose of tumor bed radiation is achieved, resulting in local control while minimizing the radiation exposure to healthy brain tissue. Furthermore, the temporal distance between surgery and adjuvant radiotherapy is decreased drastically, during which cancer cells may proliferate [15]. Correspondingly, a recent retrospective data analysis of Kahl and co-workers verified that IORT with low energy 50 kV x-rays is a safe way to apply localized brain radiotherapy after resection of brain metastases with low toxicity and excellent local control [9]. To our knowledge, data on the feasibility of intraoperative radiotherapy during awake surgery have not been published yet.

## Material and methods

In this case series, we report on five patients diagnosed with eloquently located solid metastasis or glioma. Due to the localization of the lesion, microsurgical resection was performed as an asleep-awake-asleep craniotomy with intraoperative neuromonitoring and neuronavigation. Demographical and clinical data, and surgical procedures, including the details of IORT and treatment-related complications, were analyzed. This observational retrospective study was performed according to the Declaration of Helsinki in 1995. The responsible local ethics committee of the Ludwig-Maximilians-University Munich, Germany had no ethical concerns (written request on May 24, 2022, personal communication). Patients gave informed consent to the treatment, the data analysis, and the publication of their data.

### Surgical procedures

The treatment strategies followed the recommendation of the multidisciplinary tumor board of the University Hospital Augsburg in all patients. At our local site, asleep-awake-asleep craniotomies are carried out using total intravenous anesthesia (TIVA) with propofol and remifentanil. Perioperatively, each patient standardly receives anticonvulsant prophylaxis with Levetiracetam 1000 mg. After the scalp block using Ropivacaine 0.375% and Adrenaline (1:200.000), the head of the patient is stabilized by the Mayfield clamp. During skin incision, tissue flap preparation, craniotomy, and opening of the dura, the depth of anesthesia is kept at a low level. The microsurgical tumor resection is performed with the aid of neuronavigation and neuromonitoring. Therefore, the extent of resection, in particular close to the pyramidal tract, is determined by sensory and motor evoked potentials. The analgosedation is reduced, and the entry point of the corticotomy is defined using a stimulation probe, while the patient must solve language-related tasks by correctly naming drawn objects. If stimulation of the cortex does not provoke a speech arrest, the corticotomy is started. During tumor resection, the patient must continue to name drawings correctly in the presence of the neuropsychologist. In the case of word-finding disorders or motor dysphasia, the resection must be interrupted until the speech pattern returns to normal. Simultaneously, a frozen section of the removed tissue is prepared to confirm malignancy.

**Fig. 1:**
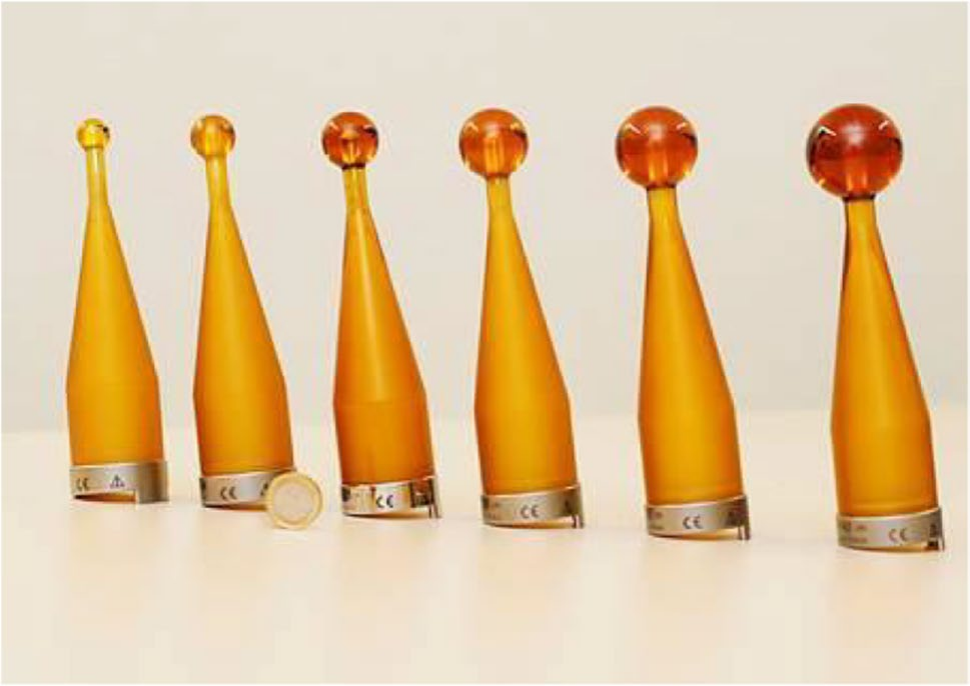
According to the resection cavity, different diameters of the spherical applicator from 1.5 to 5.0 cm are available to carry out the intraoperative irradiation

**Fig. 2:**
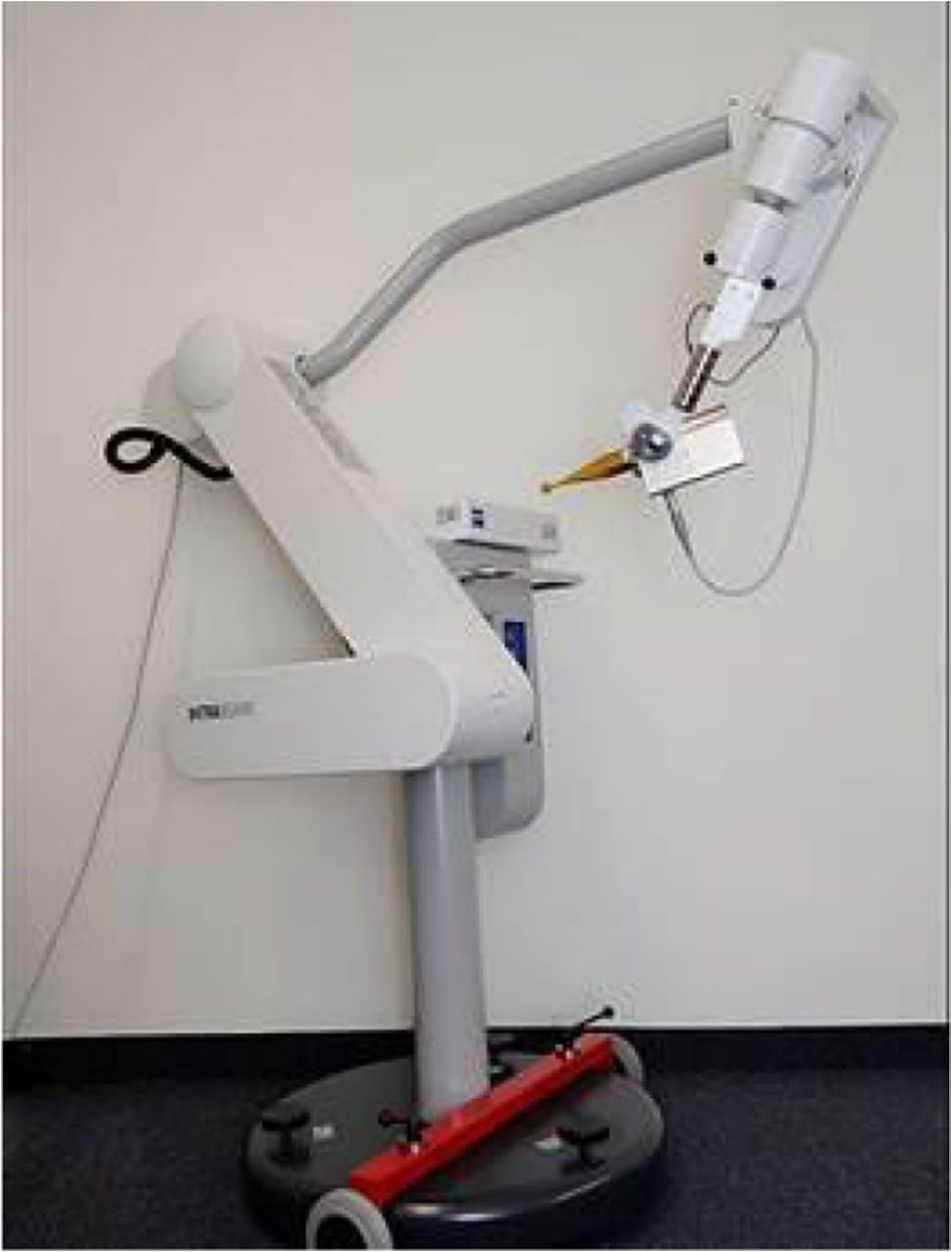
After inserting the applicator sphere, intraoperative irradiation is performed with 50-kV x-rays via an INTRABEAM system

After the tumor resection, the applicator size (Fig. 1) is chosen by the neurosurgeon according to the size of the resection cavity, and the spherical applicator was then gently inserted into the resection cavity. Attention is paid to avoid additional pressure of the applicator, which is covered by adjacent parenchyma of the cavity, on brain tissue. The intraoperative irradiation is carried out with 50-kV x-rays via an INTRABEAM system (Fig. 2) (ZEISS MEDITEC AG, Oberkochen, Germany), whereby the irradiation time is determined individually [9]. During irradiation, the analgosedation is deepened and a laryngeal mask is inserted to avoid any movement of the patient with maintenance of spontaneous breathing.

After surgery, each patient received a cumulative dose of 24 mg dexamethasone per day, which was briefly and completely tapered off within 14 days. Furthermore, within the first 48 h after the operation, brain MR imaging was performed to determine the extent of the resection.

## Results

All five patients (four male, one female; mean age: 65 ± 13.5 years) were suffering from an eloquently located solid metastasis or glioma. Before surgery, the median Karnofsky Performance Status (KPS) of the patients was 80%. Preoperatively, patients no. 1 and no. 2 suffered from intermittent speech disorders, whereas the third patient noticed behavioral changes. Patient no. 4 experienced an epileptic seizure for the first time and was suffering from pronounced dysarthria. The 5th patient developed progressive word-finding disorders. Due to the localization, the microsurgical tumor resection was performed as an awake craniotomy with neuromonitoring, neuronavigation, and neuropsychological testing. Close to Broca´s or Wernicke´s area, the tumor resection caused transitory speech disorders in some cases, which resolved ultimately in every patient. The intraoperative tumor bed irradiation was generally carried out via the INTRABEAM system with 50 KV x-rays and 20 Gy with a mean irradiation time of 16.7 ± 2.5 min. Set-up time for assembling and disassembling of the IORT system including draping with a sterile cover lasted 8 to 10 min for each patient. Either a 2.0-cm or 2.5-cm applicator sphere was applied directly into the resection cavity. Cerebral edema or relevant bleeding did not occur in any patients during the operation. After surgery, none of the patients had a new focal neurological deficit or developed a wound infection. Speech disorders improved in duration and severity, and mean postoperative KPS of the patients was 90%. The histopathological work-up showed metastasis in four cases and unexpected evidence of glioma in one case. The MR imaging of the neurocranium after surgery confirmed gross total resection in each case.

Apart from patient no. 4, who underwent adjuvant chemoradiotherapy according to the Stupp protocol, all other patients were spared from a 4-week fractional percutaneous radiation therapy with typically five individual irradiations per week. This, in turn, represents a considerable relief for the patient himself and his relatives while increasing the patient’s convenience by integrating resection and radiotherapy into one procedure [14] with no further necessity of adjuvant radiotherapy after discharge from the hospital [9]. During a mean follow-up time of 6.3 ± 2.6 months, no new neurological deficits occurred. Patient no. 5 suffered from transient word-finding disorders, although their frequencies were lower. Moreover, there were no wound infections, intracranial hemorrhage, cerebral ischemia, intracranial abscess, or other surgery and/or IORT-related complications throughout the observation period (Table 1).

**Table 1:** Pre-and postoperative data of the five patients undergoing IORT during awake craniotomy

#	Age	Sex	Pre-op status	KPS pre-op (%)	IORT Dose (Gy)	Irradiation time (min)	IORT applicator	Post-op status	KPS post-op (%)	EOR	Histopathology	Follow-up (months)
1	49	F	Motor aphasia	90	20	17.7	2.5	Temporary intermittent speech disorder	90	GTR	Metastasis of an adenocarcinoma	12
2	72	M	Intermittent motor aphasia, behavioral changes	70	20	17.7	2.5	Temporary intermittent speech disorder	80	GTR	Metastasis of an adenocarcinoma	7
3	52	M	Behavioral changes, hemiparesis of the right side	70	20	12.2	2.0	No focal neurological deficit	90	GTR	Metastasis of renal cell carcinoma	5
4	74	M	Epileptic seizure	70	20	17.9	2.5	Temporary intermittent speech disorder	90	GTR	Glioblastoma	4
5	78	M	Word-finding disorders	80	20	17.9	2.5	Intermittent word- finding disorders	90	GTR	Metastasis of a rectal adenocarcinoma	3

*#* patient, *pre-op* preoperative, *post-op* postoperative, *EOR* extent of resection, *GTR* gross total resection

### Illustrative case

**Fig. 3:**
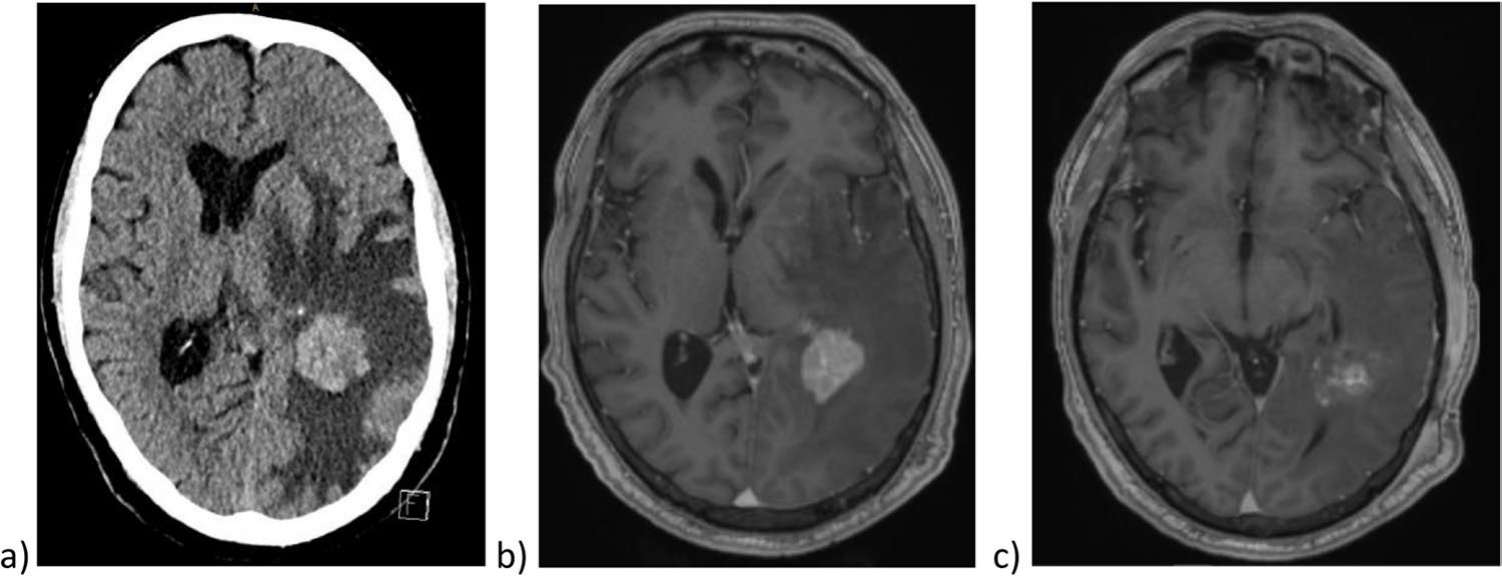
**a** Preoperative CT of the neurocranium, **b** preoperative brain MRI, and **c** MRI after surgery

A 52-year-old patient with a history of renal cell carcinoma and prostate carcinoma was suffering behavioral changes, including aggressive behavior toward strangers and increasing self-neglect. In addition, there was a latent hemiparesis of the right side with a strength grade of 4/5. His KPS was 70%. A computed tomography (CT) and magnetic resonance imaging (MRI) of the neurocranium showed a contrast-enhanced lesion left temporoparietal with central necrosis (Fig. 3a, b).

**Fig. 4:**
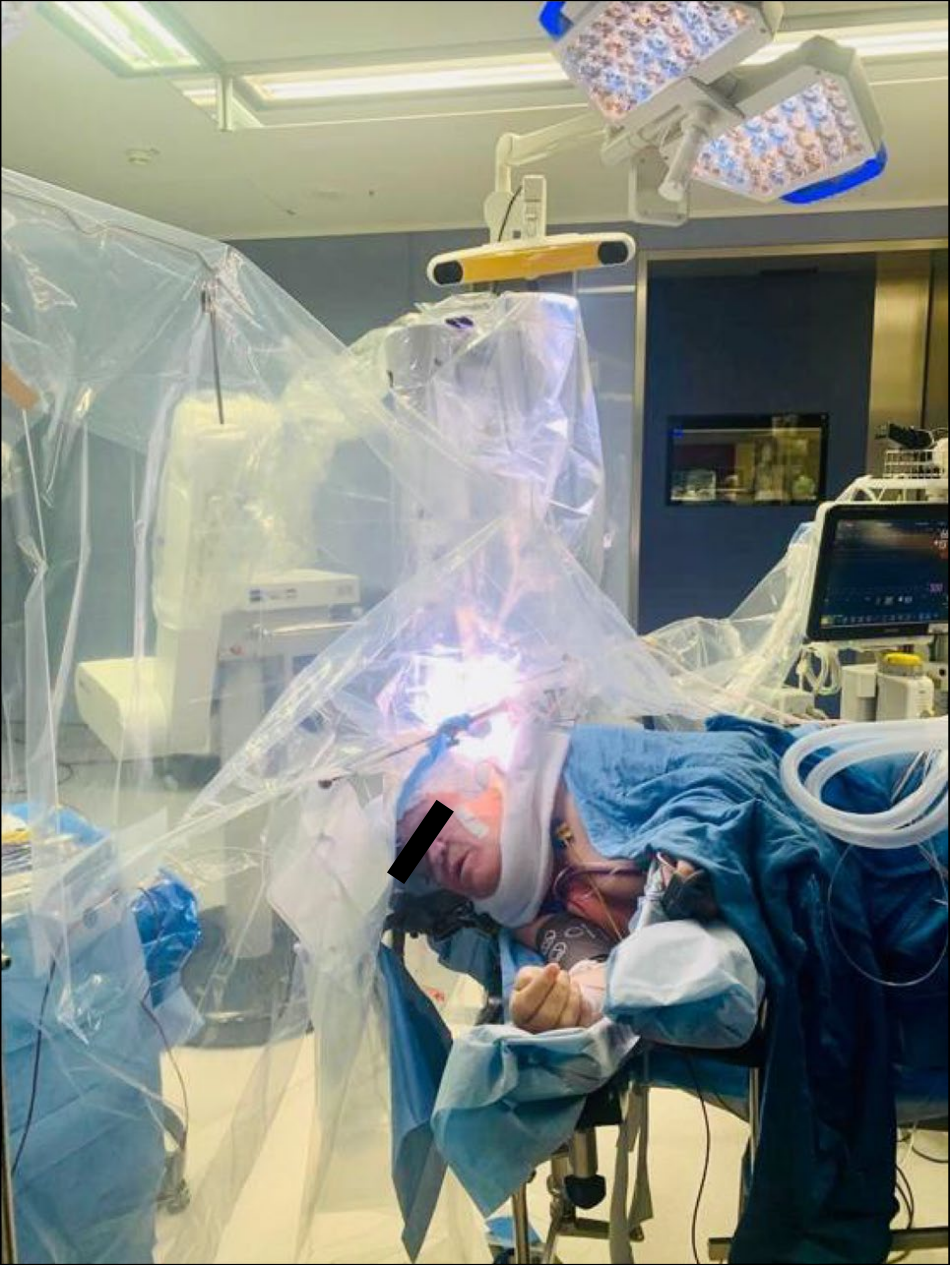
IORT following metastasis resection via an asleep-awake-asleep approach in the surgery room of the University Hospital Augsburg

Surgery was performed in an asleep-awake-asleep approach. Near the Wernicke area, the metastasis resection caused an intermittent speech arrest, which resolved after multiple interruptions of the surgical procedure. The macroscopic complete tumor resection was enabled with the additional use of neuronavigation and neuromonitoring. Subsequently, the intraoperative radiotherapy was carried out via the INTRABEAM system with 50 KV x-rays and 20 Gy (Fig. 4). Using the 2-cm applicator sphere, the irradiation lasted 12 min and 13 s.

After surgery, neither motor nor sensory dysphasia was detectable. Furthermore, there was no new sensorimotor deficit. His KPS was at 90%. The postoperative MR imaging of the neurocranium confirmed the macroscopic complete metastasis resection (Fig. 3c).

The histopathological diagnosis was a metastasis of renal cell carcinoma.

## Discussion

To our knowledge, this is the first case series of patients treated with IORT after tumor resection via an awake craniotomy. In contrast to previous publications analyzing IORT after supratentorial tumor resection under general anesthesia, we present preliminary experience on awake craniotomy and IORT, which were performed in a single procedure. Comparing the outcomes of awake craniotomy with craniotomy under general anesthesia, several studies have already demonstrated that awake craniotomy is associated with a significantly higher rate of gross total resection and a lower rate of persistent neurological deficits [5, 7, 16]. In the meta-analysis of De Witt Hamer and colleagues (2012), a relatively higher rate of early neurological deficits in the first 3 months after surgery was detected, which seems to be temporary [4, 16] shown by a higher early neurological deficit rate of 47.9% compared to a late neurological deficit rate of 6.4% [4]. Reasons for early transient neurological deficits after awake craniotomy may include resection-induced contusion, edema, or hypoperfusion. Zhang et al. (2020) favored subpial dissection and perioperative corticosteroids, similar to those used in our setting to reduce the risk of hypoperfusion and ischemia [16]. While an intraoperative seizure with an incidence of 13% is a known risk factor for procedure failure among patients undergoing an awake craniotomy, none of our patients experienced an epileptic seizure. In this context, the coordination between the surgical and anesthesia teams and the perioperative dose of anticonvulsants reduces the probability of an intraoperative epileptic seizure [11]. Considering these risk factors, the procedure and the potential complications associated with an awake craniotomy are manageable.

The anesthesiological management of asleep-awake-asleep craniotomies, in which general anesthesia or analgosedation is intermittently interrupted for neurological testing, needs specific requirements and a coordinated setting. It requires profound knowledge of neuroanesthesia including advanced airway management, to avoid complications during the procedure [13]. The anesthesiological procedures are a balancing act between overdosing anesthetics leading to impairment of respiration and alertness and underdosing directing pain, strain, and stress to the patient [12]. Pain remains a common compliant in awake craniotomies [10]. To avoid considerable stress for the patient during the surgery, the presence of a neuropsychologist is important for calming down the patient.

Our case series shows that the anesthesiologic management of non-intubated patients is critical in this combined procedure to secure a correct insertion of the applicator sphere into the resection cavity after completing the tumor resection. Therefore, after deepening the sedation without relaxation of the patient, the anesthesiologist must be able to insert the laryngeal mask, while the patient´s head remains in the Mayfield clamp. Maintaining an adequate analgosedation to keep the patient immobilized without fully intubating the patient is challenging and represents a balancing act for neuroanesthesiologists.

In patients undergoing surgical resection of eloquent brain tumors, the risk of local recurrence remains high [1]. In our case series report, local control is caused by applying the radiation source directly to the resection margin where most recurrence occurs. Moreover, there seems to be a temporal benefit of decreased delay between surgery and adjuvant treatment [14]. This is justified because waiting for wound healing is no longer necessary, which usually takes two to three weeks before the percutaneous radiation starts. Based on the current literature, the observed local control rate of the resection cavity after IORT averages between 75 and 88% [3, 7, 9]. In addition, the extent of the resection was found to be the significant predictor of local control following IORT treatment of the resection cavity in their patient population [3].

Awake craniotomy can achieve higher rates of macroscopically complete tumor resection in eloquent brain areas significantly affecting local tumor mass control after IORT.

## Limitations

This case series is limited by its design and the short follow-up time span. To deliver a profound statement, middle- and long-term results with a higher case load are needed. We are planning a prospective observational trial in the near future to evaluate the advantages of this combined procedure by verifying the high one-year local control and low incidence of complications.

## Conclusion

According to our preliminary data, the combination of awake craniotomy and intraoperative radiotherapy seems feasible and safe.

## References

[1] Bilger A, Bretzinger E, Fennell J, Nieder C, Lorenz H, Oehlke O, Grosu AL, Specht HM, Combs SE (2018). Local control and possibility of tailored salvage after hypofractionated stereotactic radiotherapy of the cavity after brain metastases resection. Cancer Med.

[2] Cifarelli CP, Jacobson GM (2021). Intraoperative radiotherapy in brain malignancies: indications and outcomes in primary and metastatic brain tumors. Front Oncol.

[3] Cifarelli CP, Brehmer S, Vargo JA, Hack JD, Kahl KH, Sarria-Vargas G, Giordano FA (2019). Intraoperative radiotherapy (IORT) for surgically resected brain metastases: outcome analysis of an international cooperative study. J Neurooncol.

[4] De Witt Hamer PC, Robles SG, Zwinderman AH, Duffau H, Berger MS (2012). Impact of intraoperative stimulation brain mapping on glioma surgery: a meta-analysis. J Clin Oncol.

[5] Esenou C, Rincon-Torroella J, ReFaey K, Lee YM, Nangiana J, Vivas-Buitrago T, Quinones-Hinojosa A (2017). Awake craniotomy vs craniotomy under general anesthesia for perirolandic gliomas: evaluating perioperative complications and extent of resection. Neurosurgery.

[6] Fuentes R, Osorio D, Hernandez JE, Simancas-Racines D, Martinez-Zapata MJ, Cosp XB (2018) Surgery versus stereotactic Radiotherapy for people with single or solitary brain metastasis. Cochrane Database Syst. Rev. 8(8): CD012086. 10.1002/14651858.CD12086.pub 10.1002/14651858.CD012086.pub2 PMC6513097 30125049

[7] Gerritsen JKW, Vietor CL, Rizopoulos D, Schouten JW, Klimek M, Dirven CMF, Vincent AJPE (2019). Awake craniotomy versus craniotomy under general anesthesia without surgery adjuncts for supratentorial glioblastoma in eloquent areas: a retrospective matched case-control study. Acta Neurochir.

[8] Giordano FA, Brehmer S, Mürle B, Welzel G, Sperk E, Keller A, Abo-Maydan Y, Scherzinger E, Clausen S, Schneider F, Herskind C, Glas M, Seiz-Rosenhagen M, Groden C, Hänggi D, Schmiedek P, Emami B, Souhami L, Petrecca K, Wenz F (2019). Intraoperative Radiotherapy in Newly Diagnosed Glioblastoma (INTRAGO): an open-label, dose-escalation phase I/II trial. Neurosurgery.

[9] Kahl KH, Balagiannis N, Höck M, Schill S, Roushan Z, Shiban E, Müller H, Grossert U, Konietzko I, Sommer B, Maurer CJ, Berlis A, Heidecke V, Janzen T, Stüben G (2021). Intraoperative radiotherapy with low-energy x-rays after neurosurgical resection of brain metastases — an Augsburg University Medical Center experience. Strahlenther Onkol.

[10] Kulikov A, Lubnin A (2018). Anesthesia for awake craniotomy. Curr Opin Anaesthesiol.

[11] Nossek E, Matot I, Shahar T, Barzilai O, Rapoport Y, Gonen T, Sela G, Grossmann R, Korn A, Hayat D, Ram Z (2013). intraoperative seizures during awake craniotomy: incidence and consequences. Neurosurgery.

[12] Seemann M, Zeck N, Graf B, Hansen E (2015). Anesthesiological management of awake craniotomy: asleep-awake-asleep technique without sedation. Anaesthesist.

[13] Stevanovic A, Rossaint R, Veldeman M, Bilotta F, Coburn M (2016). Anaesthesia management for awake craniotomy: systematic review and meta-analysis. PLoS ONE.

[14] Vargo JA, Sparks KM, Singh R, Jacobson GM, Hack JD, Cifarelli CP (2018). Feasibility of dose escalation using intraoperative radiotherapy following resection of large brain metastases compared to postoperative stereotactic radiosurgery. J Neurooncol.

[15] Ylanan AMD, Pascual JSG, Cruz-Lim AMD, Ignacio KHD, Canal JPA, Khu KJO (2021). Intraoperative radiotherapy for glioblastoma: a systematic review of techniques and outcomes. J Clin Neurosci.

[16] Zhang JJY, Lee KS, Voisin MR, Hervey-Jumper SL, Berger MS, Zadeh G (2020) Awake craniotomy for resection of supratentorial glioblastoma: a systematic review and meta- analysis**.** Neurooncol Adv. 2(1): vdaaa11. 10.1093/noajnl/vdaa111 10.1093/noajnl/vdaa111 PMC7542985 33063012

